# Association of transverse sinus stenosis and dominant venous sinus drainage pattern with chronic migraine: A matched case-control study

**DOI:** 10.1097/MD.0000000000048710

**Published:** 2026-07-24

**Authors:** Xianrong Xiang, Shiyu Zhu, Xichang Liu

**Affiliations:** a Department of Neurology, Affiliated Renhe Hospital of China Three Gorges University, Yichang, China; b Department of Neurology, Xiling Branch of Yichang Central People’s Hospital, Yichang, China.

**Keywords:** bilateral transverse sinus, chronic migraine, MRI, venous sinus dominance pattern

## Abstract

Recent studies have indicated that transverse sinus stenosis (TSS) is an imaging feature in patients with chronic migraine (CM). However, the clinical significance of this imaging finding in CM remains unclear. Moreover, the role of the dominant venous sinus drainage pattern in CM remains to be elucidated. This matched case-control study aimed to investigate the associations of TSS and venous sinus dominance pattern with CM. This study included 94 patients with CM and 94 sex- and age-matched controls. All included patients underwent magnetic resonance venography. Dominant venous sinus drainage pattern (right-dominant, left-dominant, or equally dominant) was quantitatively assessed by consultant neuroradiologists. Venous sinus stenosis was determined using the combined conduit score (1–8) and was further classified into 3 categories: bilateral stenosis, unilateral stenosis, and no stenosis. No significant difference was found in the dominant venous sinus drainage pattern between CM and control patients. Multivariable logistic regression analysis demonstrated a strong positive correlation between TSS and CM (bilateral: odds ratio = 6.60, 95% confidence interval: 2.30–18.91; unilateral: odds ratio = 2.41, 95% confidence interval: 1.01–5.26; all *P* < .05). Venous sinus dominance pattern did not differ between CM patients and controls. In contrast, TSS (particularly bilateral) was significantly associated with CM. These findings suggest a potential association between impaired cerebral venous outflow and migraine chronification; however, causality cannot be inferred.

## 1. Introduction

Migraine is a neurovascular disorder characterized by episodic attacks of intense headache. It affects 14.0% of the population, ranking as the second-leading cause of disability worldwide and the primary cause among young women.^[1,2]^ Based on monthly headache frequency, migraine is classified as episodic migraine (<15 headache days per month) or chronic migraine (CM; ≥15 headache days per month for ≥3 months).^[3]^ Severe pain not only causes significant functional impairment in daily life and occupational productivity but also compromises the patient’s psychological well-being.^[4]^ These features are more pronounced in patients with CM; however, the structural or physiological factors that may contribute to migraine chronification remain incompletely understood.

The dural venous sinuses receive trigeminal afferent innervation and constitute a key component of the trigeminovascular system implicated in migraine pathogenesis.^[5,6]^ Transverse sinus stenosis (TSS), the most common form of venous sinus stenosis, is defined as focal or segmental luminal narrowing, which may impede cerebral venous outflow and disrupt intracranial pressure homeostasis.^[7,8]^ Some evidence suggests that impaired venous drainage may contribute to migraine chronification. The underlying mechanism may be related to elevated venous sinus pressure accompanying TSS. Elevated venous sinus pressure secondary to TSS may result in sustained mechanical stimulation of pain-sensitive venous structures, potentially increasing headache frequency.^[9]^

Recent clinical studies have suggested that TSS is frequently observed on brain magnetic resonance imaging (MRI) in clinical practice among patients with CM.^[10–12]^ Approximately 47.5% of patients with CM exhibit varying degrees of TSS.^[13]^ Compared with other patients with migraine, those with transverse sinus hypoplasia are associated with greater analgesic use, higher anxiety levels, and elevated systolic blood pressure. These factors have been reported to be associated with migraine chronification.^[12]^ In addition, a subset of patients with CM meet criteria for idiopathic intracranial hypertension (IIH) without papilledema, a condition frequently accompanied by bilateral TSS. However, whether TSS is a risk factor for CM or a comorbidity, as well as its role in intracranial pressure in CM, remains unclear.

Beyond TSS, variations in dominant venous sinus drainage may also influence local venous hemodynamics. Previous studies have reported that only 47% of venous sinus drainage patterns show equal dominance in a generalized population, while the remainder demonstrate lateral dominant drainage, either right or left.^[14]^ Although the prevalence of venous sinus dominance is well characterized in the general population, its distribution and clinical relevance in patients with CM remain unclear. Prior studies have largely evaluated TSS in isolation, without accounting for dominant venous drainage as a potential confounder. Accordingly, the independent and combined associations of TSS and venous sinus dominance with CM warrant systematic investigation.

## 2. Materials and methods

This retrospective study was approved by the Ethics Committee of The First College of Clinical Medicine, China Three Gorges University (Approval No: 2024-320-01), which granted a waiver of informed consent as the research involved the analysis of existing anonymous clinical data.

The minimum required sample size was estimated using a two-sided α of 0.05 and a statistical power (1 − β) of 0.80. Based on prior studies reporting an odds ratio (OR) of approximately 3.5 for the association between TSS and CM, the required total sample size was 160 (comprising 80 cases and 80 matched controls).^[13,15]^

Data were collected between January 2020 and August 2024. Participants were selected based on specific inclusion criteria, which included patients diagnosed with CM, according to The International Classification of Headache Disorders, 3rd edition (beta version), and who underwent unenhanced magnetic resonance venography. Control subjects were selected from non-headache patients. Exclusion criteria encompassed other diseases that may produce headaches, such as tumors, intracranial organic diseases, cerebrovascular disease, or cerebral venous sinus thrombosis, and incomplete clinical data that may affect the study outcome for patients.

The dominant venous sinus drainage patterns and the degree of venous sinus stenosis were independently evaluated at the imaging workstation by a senior attending neurologist. The classification standard of dominant drainage includes equal dominance, left lateral dominant drainage, and right lateral dominant drainage. Dominance was quantitatively defined as a diameter disparity exceeding 20% between contralateral sinuses.^[16]^ Vascular stenosis was assessed using the combined conduit score (CCS).^[8]^ The CCS quantifies stenosis severity from the torcular to the distal sigmoid sinus using a 0 to 4 scale: 0 = discontinuity, 1 = severe stenosis/hypoplasia (<25% lumen diameter), 2 = moderate stenosis (25%–50%), 3 = mild stenosis (50%–75%), and 4 = no significant narrowing (75%–100%). CCS is the sum of the highest stenosis scores from both sides, with stenosis defined as CCS <3. Concurrently, bilateral scores were also documented independently.^[8]^ Stenosis was also defined as bilateral, unilateral, or absent.

## 3. Statistics

Data analysis was performed using R software (version 4.3.2; R Foundation for Statistical Computing). A 1:1 nearest neighbor matching on age and sex was performed using the MatchIt package in R (R Foundation for Statistical Computing). The Shapiro–Wilk test was used to assess the normality of continuous variables. Continuous variables with normal distribution are presented as mean ± standard deviation, while categorical variables are expressed as frequencies (percentages). Differences between groups were compared using the *t* test or Mann–Whitney *U* test for continuous variables and the χ^2^ test or chi-squared test for categorical variables. Univariate conditional logistic regression was first used to assess the association between each variable and CM. Variables with *P* <.10 in univariate analysis were considered for inclusion in the multivariable conditional logistic regression model to avoid premature exclusion of potentially relevant confounders. Key exposure variables were retained based on a priori clinical relevance. Results are presented as OR with 95% confidence intervals (CI). Statistical significance was defined as *P* <.05.

## 4. Results

### 4.1. Demographic and cerebral venous characteristics after matching

**Figure 1.**
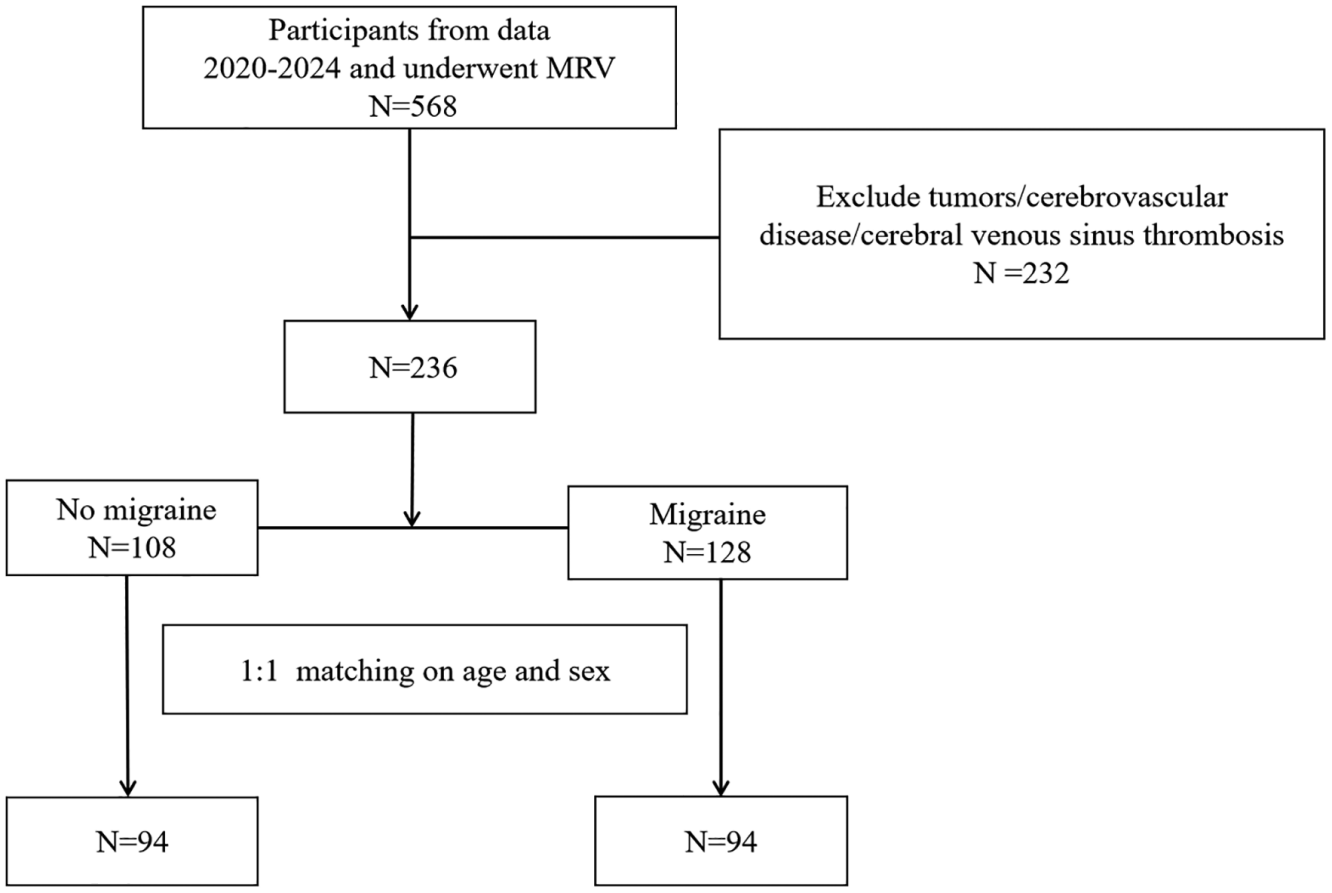
Flow diagram of the study. MRV = magnetic resonance venography.

This study included 94 patients with CM and 94 age- and sex-matched healthy controls. No significant differences were observed between the 2 groups in these matching variables (both *P* > .05). The detailed flowchart of participant inclusion and exclusion is shown in Figure 1. The demographic characteristics and cerebral venous characteristics are shown in Table 1. Dural venous sinus dominance distributions did not differ significantly between CM patients and controls. Similarly, no associations were observed for body mass index, education status, cardiopathy, or diabetes mellitus (all *P* > .05). In contrast, CCS was significantly different between the CM and control groups (*P* < .001). After stratifying by CCS grade, the association between stenosis type and CM remained statistically significant (*P* < .05).

**Table 1:** Characteristics of the study population.

Characteristics	Controls (94)	CM (94)	*P* value
Age, mean (SD)	44.47 (11.62)	45.35 (12.04)	.61
Female, n (%)	62 (66.0)	62 (66.0)	1
BMI, n (%)			.576
Normal	68 (72.3)	69 (73.4)	
Overweight	20 (21.3)	22 (23.4)	
Obese	6 (6.4)	3 (3.2)	
Educational status, n (%)			.552
Low (primary/middle)	48 (51.1)	46 (48.9)	
Medium (high school)	15 (16.0)	11 (11.7)	
High (college/university)	31 (33.0)	37 (39.4)	
Hypertension, n (%)	22 (23.4)	15 (16.0)	.271
DM, n (%)	6 (6.4)	3 (3.2)	.494
Cardiopathy, n (%)	4 (4.3)	3 (3.2)	1
CCS, mean (SD)	6.44 (1.94)	4.70 (2.57)	**<.001** *
Stenosis type, n (%)			**.046** *
Absent	53 (60.2)	38 (43.2)	
Unilateral	28 (31.8)	35 (39.8)	
Bilateral	7 (8.0)	15 (17.0)	
Drainage, n (%)			.212
E	40 (42.6)	30 (31.9)	
R	43 (45.7)	55 (58.5)	
L	11 (11.7)	9 (9.6)	

Data are expressed as mean ± SD and counts and percentages. The *t* test or Mann–Whitney *U* (Wilcoxon) test and χ^2^ test were performed.

Bold values indicate statistically significant differences between the two groups (*P* < .05).

BMI = body mass index, CM = chronic migraine, CCS = combined conduit score, DM = diabetes mellitus, E = equal dominant drainage, L = left lateral dominant drainage, R = right lateral dominant drainage, SD = standard deviation.

* *P* < .05.

### 4.2. Univariable and multivariable logistic regression analysis in CM and controls

**Figure 2.**
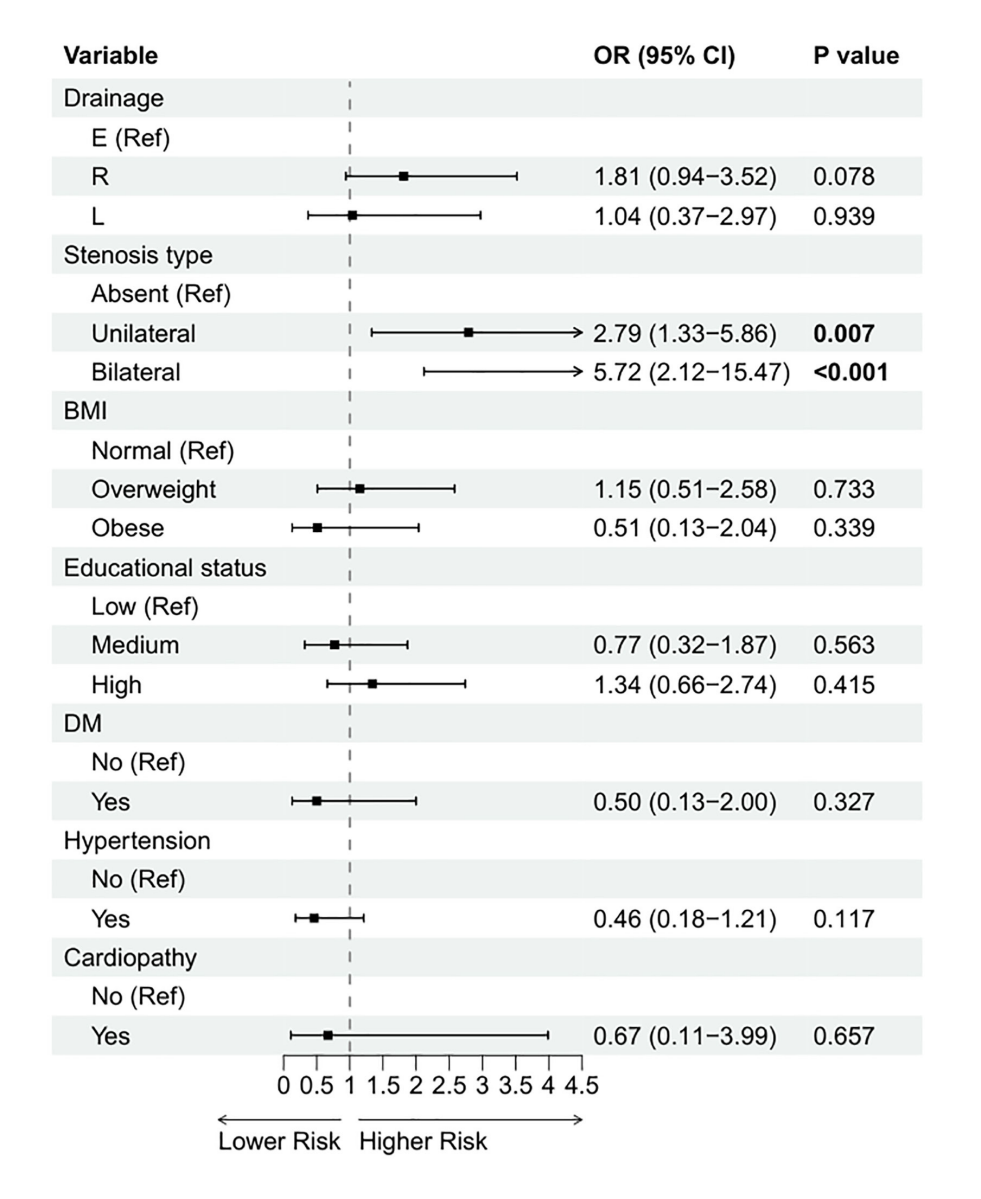
Conditional logistic regression for univariable analysis. BMI = body mass index, CI = confidence interval, DM = diabetes mellitus, E = equal dominant drainage, L = left lateral dominant drainage, OR = odds ratio, R = right lateral dominant drainage, Ref = reference group.

**Figure 3.**
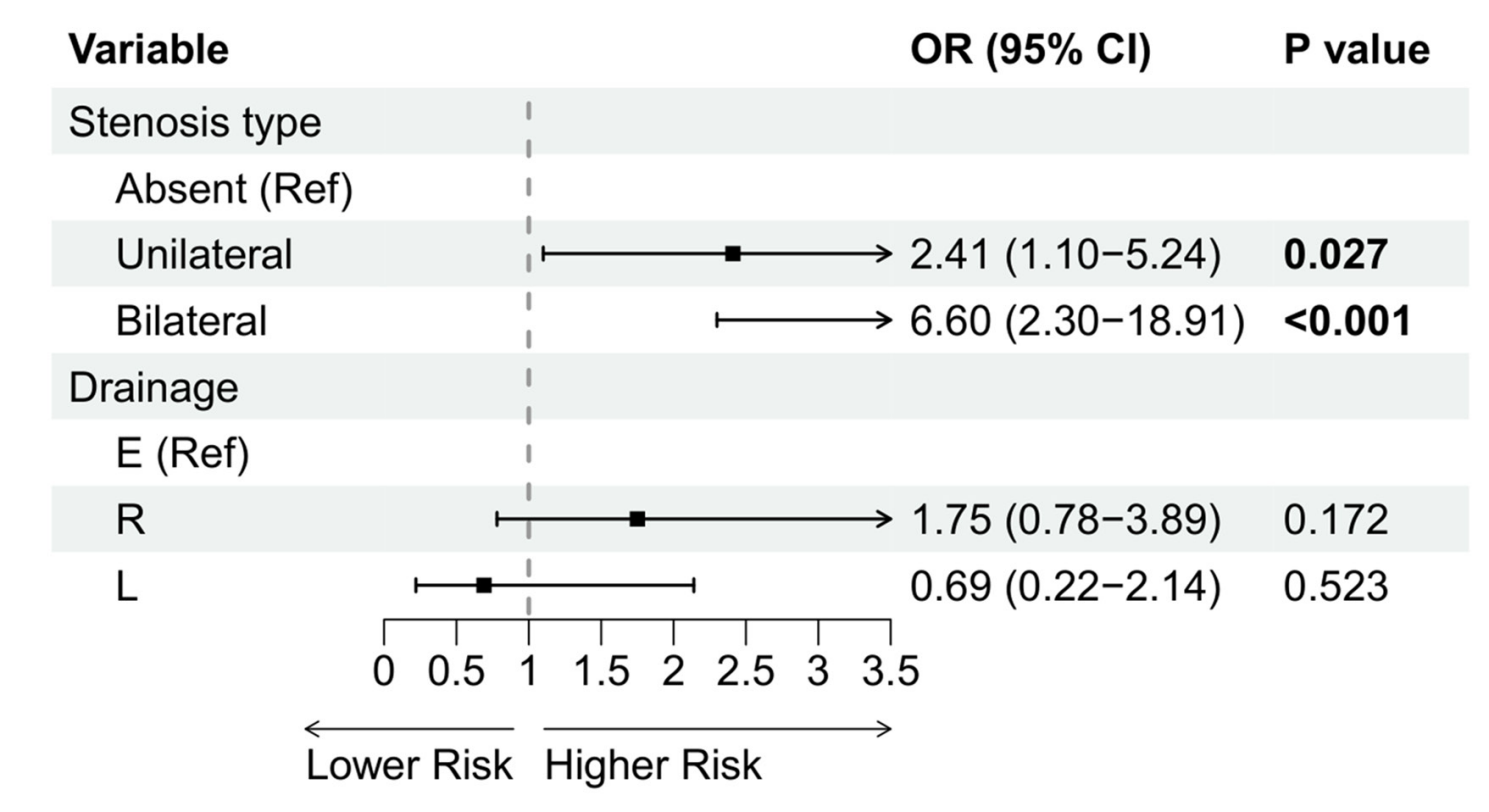
Conditional logistic regression for multivariable analysis. CI = confidence interval, E = equal dominant drainage, L = left lateral dominant drainage, OR = odds ratio, R = right lateral dominant drainage, Ref = reference group.

Univariate and multiple conditional logistic regression analyses were used to assess the associations between CM and the following factors: TSS and the dominant venous sinus drainage pattern. The results of the univariate and multivariate analyses are shown in Figures 2 and 3, respectively. In univariate conditional logistic regression with co-dominant as reference, no venous pattern showed a significant association with migraine status (R: OR = 1.81, 95% CI = 0.94–3.52; L: OR = 1.04, 95% CI = 0.37–2.97; both *P* > .05). There were also no associations observed for body mass index, education status, cardiopathy, or diabetes mellitus (all *P* > .05). Univariate regression analysis showed that bilateral TSS (OR = 5.73, 95% CI = 2.12–15.47, *P* < .05) and unilateral TSS (OR = 2.79, 95% CI = 1.33–5.86, *P* < .05) were associated with CM. In conditional logistic regression for multivariable analysis, bilateral (OR = 6.60, 95% CI = 2.3–18.91, *P* < .05) and unilateral TSS (OR = 2.41, 95% CI = 1.10–5.42, *P* < .05) demonstrated a significant independent association with CM. For drainage patterns, neither L-type nor R-type drainage exhibited significant associations compared to E-type.

## 5. Discussion

In this study, we found no significant association between dominant venous sinus drainage pattern and CM. In contrast, both bilateral and unilateral TSS were significantly associated with CM after matching and multivariable adjustment. These findings suggest that TSS is more likely to represent a structural correlate associated with CM, whereas venous sinus dominance appears to reflect a normal anatomical variation without clinical relevance in this context.

According to previous studies, right-sided transverse sinus dominance is observed in approximately 47% to 49% of the general population, whereas left-sided dominance is considerably less frequent, occurring in 13% to 15% of individuals.^[14,17]^ In our study, a similar distribution pattern was observed among patients with CM, with right-sided dominance being the most common (57.3%) and left-sided dominance the least frequent (5.6%). However, no statistically significant difference in venous sinus dominance pattern was observed between patients with CM and healthy controls. These findings suggest that venous sinus dominance pattern is more likely to represent a normal anatomical variation rather than a clinically relevant factor associated with CM.

The reported prevalence of TSS among patients with CM varies across studies. Bono et al observed bilateral stenosis in 49% of TSS cases within a mixed cohort of CM and chronic tension-type headache.^[11]^ De Simone et al reported an overall TSS prevalence of 92.8% among patients with refractory CM, including 34% with bilateral involvement, whereas Favoni et al documented a lower prevalence of 47.5%, with bilateral stenosis in 17.5% of cases.^[13,15]^ In our study, the overall prevalence of TSS was 56.8%, with bilateral stenosis present in 17.0% of patients, placing our findings within the range of prior reports. Importantly, after adjustment for age and sex, multivariable logistic regression analysis demonstrated that TSS was independently associated with CM. However, the directionality of this association remains uncertain. Given the retrospective design, causality cannot be inferred. The association between CM and TSS may involve elevated intracranial pressure secondary to venous outflow obstruction and subsequent activation of the trigeminovascular system.^[18]^ The high prevalence of bilateral TSS in IIH, a disorder commonly presenting with migraine-like headache, is consistent with this hypothesis.^[7,19]^ Taken together, these findings suggest that assessment of TSS may have potential clinical relevance in the evaluation of patients with migraine.

This study has several limitations that should be considered. First, bilateral TSS, a key imaging feature under investigation, is also a well-established radiological marker of IIH, which exhibits clinical overlap and potential comorbidity with migraine.^[7]^ Because cerebrospinal fluid pressure was not routinely measured in this retrospective cohort, we cannot fully exclude the possibility that some patients with atypical IIH were misclassified as having CM.^[20]^ Prior studies suggest that combining multiple MRI features, including TSS, empty sella, and optic nerve sheath distension, improves specificity for IIH.^[21]^ In our cohort, no additional MRI findings suggestive of IIH were identified, making substantial misclassification less likely. Second, although age and sex were controlled, residual confounding may persist. Variables such as migraine duration, attack frequency, disease severity, and use of preventive or acute medications were not included in the multivariable model. These factors may influence intracranial hemodynamics or reflect disease chronicity and could therefore affect the observed association. In addition, TSS was assessed exclusively by magnetic resonance venography without complementary hemodynamic measurements. Therefore, we could not determine whether the observed stenoses were functionally significant or represented anatomical variants, which should be considered when interpreting the clinical implications of our findings. Finally, the retrospective single-center design and limited sample size may limit generalizability. Future prospective multicenter studies are needed. The incorporation of digital subtraction angiography with simultaneous assessment of cerebral venous hemodynamics would enable a more comprehensive evaluation of both morphological and functional venous changes. In addition, well-designed prospective interventional studies are required to clarify whether TSS plays a causal role in migraine or represents a secondary phenomenon.

## 6. Conclusion

In this matched case-control study, the distribution of dominant venous sinus drainage patterns in patients with CM did not differ significantly from that of matched controls, suggesting that these anatomical variations are unlikely to be independently associated with CM. In contrast, TSS, particularly bilateral TSS, was independently associated with CM. These findings support a potential link between altered cerebral venous outflow and migraine chronification. Prospective longitudinal studies incorporating hemodynamic assessment are warranted to clarify the temporal and mechanistic relationships underlying this association.

## Author contributions

**Conceptualization:** Xichang Liu.

**Data curation:** Xianrong Xiang.

**Formal analysis:** Xianrong Xiang.

**Investigation:** Xianrong Xiang, Shiyu Zhu.

**Project administration:** Xianrong Xiang.

**Software:** Xianrong Xiang.

**Methodology:** Xichang Liu.

**Resources:** Shiyu Zhu.

**Validation:** Shiyu Zhu.

**Visualization:** Shiyu Zhu.

**Writing – original draft:** Xianrong Xiang.

**Writing – review & editing:** Xichang Liu.
